# Country-specific approaches to latent tuberculosis screening targeting migrants in EU/EEA* countries: A survey of national experts, September 2019 to February 2020

**DOI:** 10.2807/1560-7917.ES.2022.27.12.2002070

**Published:** 2022-03-24

**Authors:** Ioana Margineanu, Kieran Rustage, Teymur Noori, Dominik Zenner, Christina Greenaway, Manish Pareek, Onno Akkerman, Sally Hayward, Jon S Friedland, Delia Goletti, Ymkje Stienstra, Sally Hargreaves, on behalf of the ESGITM/ESGMYC Study Groups

**Affiliations:** 1 University of Groningen, University Medical Centre Groningen, Groningen, the Netherlands; 2 The Migrant Health Research Group, Institute for Infection and Immunity, St George’s, University of London, London, UK; 3 European Centre for Disease Prevention and Control (ECDC), Stockholm, Sweden; 4 Queen Mary’s, University of London, London, UK; 5 McGill University, Department of Medicine, Montreal, Canada; 6 University of Leicester, Leicester, UK; 7 Faculty of Public Health and Policy, London School of Hygiene & Tropical Medicine, London, United Kingdom; 8 Translational Research Unit, Department of Epidemiology and Preclinical Research, National Institute for Infectious Diseases L. Spallanzani, Rome Italy; 9 The ESGITM/ESGMYC Study Groups are acknowledged at the end of the article

**Keywords:** latent tuberculosis infection, tuberculosis, migrant, health-service delivery, health policy, Europe

## Abstract

**Background:**

Migrants in low tuberculosis (TB) incidence countries in the European Union (EU)/European Economic Area (EEA) are an at-risk group for latent tuberculosis infection (LTBI) and are increasingly included in LTBI screening programmes.

**Aim:**

To investigate current approaches and implement LTBI screening in recently arrived migrants in the EU/EEA and Switzerland.

**Methods:**

At least one TB expert working at a national level from the EU/EEA and one TB expert from Switzerland completed an electronic questionnaire. We used descriptive analyses to calculate percentages, and framework analysis to synthesise free-text responses.

**Results:**

Experts from 32 countries were invited to participate (30 countries responded): 15 experts reported an LTBI screening programme targeting migrants in their country; five reported plans to implement one in the near future; and 10 reported having no programme. LTBI screening was predominantly for asylum seekers (n = 12) and refugees (n = 11). Twelve countries use ‘country of origin’ as the main eligibility criteria. The countries took similar approaches to diagnosis and treatment but different approaches to follow-up. Six experts reported that drop-out rates in migrants were higher compared with non-migrant groups. Most of the experts (n = 22) called for a renewed focus on expanding efforts to screen for LTBI in migrants arriving in low-incidence countries.

**Conclusion:**

We found a range of approaches to LTBI screening of migrants in the EU/EEA and Switzerland. Findings suggest a renewed focus is needed to expand and strengthen efforts to meaningfully include migrants in these programmes, in order to meet regional and global elimination targets for TB.

## Introduction

Recent estimates place the current global latent tuberculosis infection (LTBI) prevalence at 1.3 billion people (roughly 25% of the world population) [1-3]. Approximately 5–15% of LTBI infections progress to active disease, but this could be higher for risk groups [4,5], which include migrants.

Recently arrived migrants (defined as foreign born individuals) moving from high incidence tuberculosis (TB) areas are thought to be an at-risk group for LTBI and progression to active disease, particularly within the first 5 years of arrival [6,7]. In the European Union (EU)/European Economic Area (EEA) countries, the majority of active TB cases in migrants are reactivated LTBI acquired in the country of origin, so migrant populations face a disproportionate burden of active disease [6,7].

Guidance from the European Centre for Disease Prevention and Control (ECDC) [8] recommends offering LTBI screening to all migrants from high-burden TB countries on arrival to low-incidence countries. The World Health Organization (WHO) [6] has published guidelines, but it is not clear to what extent these guidelines are followed [9] and how EU/EEA countries screen and treat recently arrived migrants for LTBI, so there is an urgent need to explore lessons learned and to share best practice [10,11].

One recent systematic review published in 2020 highlighted that in countries that focus on identifying migrants who are LTBI positive on arrival, only 54% of migrants with a positive LTBI test complete treatment [4], raising questions about how successful these programmes are in including migrant patients and engaging them in the screening and treatment pathway. Evolving treatment options, including shorter treatment regimens, could noticeably improve delivery of LTBI programmes in at-risk groups. Particular efforts [12] are now being made to engage underserved and diverse groups, including migrants, in personalised and culturally appropriate TB treatment schemes, which are equally applicable to LTBI programmes and could improve treatment outcomes particularly when combined with newer treatment options.

Current research indicates that the effectiveness and cost-effectiveness of LTBI screening and treatment are limited but could be improved by strengthening care cascades and tailoring screening to specific groups [10,13,14]. However, it is unclear to what extent EU/EEA countries are adapting services to specifically respond to the needs of migrants and many questions about optimal approaches remain [15]. Therefore, we developed an electronic questionnaire survey targeting national TB experts from all EU/EEA countries and Switzerland. This survey gathered information on current approaches to LTBI screening of recently arrived migrants and perspectives around respective policies and practices across the Region.

## Methods

### Approach to questionnaire development

We developed an electronic questionnaire containing structured and open-ended questions about country-specific LTBI screening policies for recently arrived migrants in the EU/EEA and Switzerland (at the time of conducting this research, the UK was still a part of the EU/EEA). Switzerland was included because the country has hosted many refugees since 2015. Previous studies have successfully gathered data from national experts [16]. For survey development, we sought expert input from members of the European Society of Clinical Microbiology and Infectious Diseases (ESCMID) Study Group for Infections in Travellers and Migrants (ESGITM) and the Study Group for Mycobacterial Infections (ESGMYC). The questionnaire was designed to take 15 min to complete and comprised five sections dealing with different aspects of LTBI management: current approach, policies, and practice for LTBI screening in recently arrived migrants; approaches to diagnosis, treatment, and follow-up; and innovations and next steps in delivery of LTBI screening of migrants. The survey contained open-ended questions to gain broader perspectives from across the region on the future of LTBI policies and practices.

For the purpose of this research, we defined recently arrived migrants as foreign-born persons living in the host country for fewer than 10 years, refugees as individuals granted asylum in the host country, and asylum seekers as individuals waiting for a decision on their asylum application in the host country. These definitions were listed at the beginning of the questionnaire (Supplement S1).

The questionnaire was piloted with two TB experts (part of the ESGITM working group) and their input was used to refine and improve the final questionnaire. These experts were excluded from the analysis. The questionnaire was written in English and translated upon request.

### Approach and data analysis

We approached one expert from each of the 32 EU/EEA countries and Switzerland. We drew on the expertise of members of the ESGITM and ESGMYC network to help us identify key TB/LTBI experts in each country and identify the expert either responsible or with direct knowledge of a current national TB/LTBI programme. If the expert initially approached was not directly involved in a national TB/LTBI programme, we identified the correct expert by consulting with other experts within the country, by performing digital searches in PubMed to identify the senior and first authors of country-specific TB/LTBI publications, or by reading official national websites (e.g., Ministries of Health official websites, Public Health or National TB Programmes websites). We aimed to identify one expert per country working at the national level within the field of TB, for example, at the Ministry of Health, a public health institution, or a national TB programme or an equivalent. Contact was made via email and with telephone follow-up calls when needed. Experts were contacted between September 2019 and February 2020. Those who agreed to participate were sent the survey via email. A first reminder was sent after 10 days, and subsequent reminders each consecutive week were sent when needed. The experts returned the completed survey by email.

We contacted one expert from each of the following 32 countries : Austria, Belgium, Bulgaria, Croatia, Cyprus, Czech Republic, Denmark, Estonia, Finland, France, Germany, Greece, Hungary, Iceland, Ireland, Italy, Latvia, Liechtenstein, Lithuania, Luxembourg, Malta, the Netherlands, Norway, Poland, Portugal, Romania, Slovakia, Slovenia, Spain, Sweden, Switzerland, and the United Kingdom (UK).

Data were extracted from the completed questionnaires by one researcher (IM) in Microsoft Excel, and the process was repeated by a second researcher (KR). A framework analysis was conducted to synthesise free-text responses to the open-ended questions and responses were organised in Microsoft Excel. Data on TB incidences of countries were extracted from WHO figures [17].

### Ethical statement

Raw data are kept on a secure server located at the University Medical Center Groningen (Rijksuniversiteit Groningen). The study meets all the university’s General Data Protection Regulation (GDPR) requirements. No identifiable data are contained in any published materials. No individual ethical approval was needed as the experts contacted agreed to complete the questionnaire.

## Results

### Survey response

Of the 32 experts contacted, 30 completed the survey. We were unable to ascertain a response from Hungary. The contacted German expert reported back that policies for LTBI differed between the 16 different federal states. Three experts were working directly for Ministries of Health, 18 were affiliated with national TB programmes or institutes, and nine were working within national public health or communicable diseases institutions. Detailed information on the expert group and their expertise can be found in Supplement S2. The survey was translated into Romanian.

### Implementation of LTBI screening programmes including migrants

**Figure 1:**
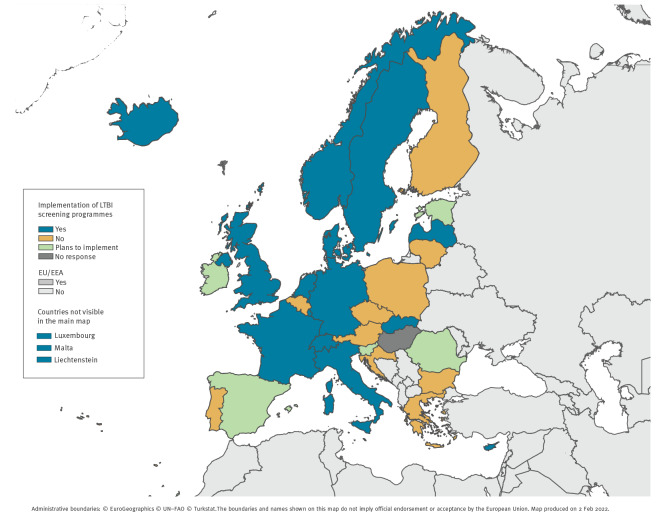
Implementation of latent tuberculosis infection screening programmes, European Union/European Economic Area countries and Switzerland, September 2019–February 2020 (n = 32)

Of the 30 experts, 15 reported that their country offered recently arrived migrants LTBI screening, five reported that their country had plans to implement LTBI screening for migrants, and 10 reported that their country had no plans to implement LTBI screening for migrants (Figure 1).

The 10 experts who reported no LTBI screening for recently arrived migrants gave divergent responses in terms of the utility of screening and future directions. Seven of these 10 experts did not report any intention to expand screening, citing that there were too few migrants in the country to make it worthwhile. In addition, one of the experts, from a country with a TB incidence of 8.4 per 100,000 cases, cited a lack of evidence of cost effectiveness. Other experts in countries not performing screening programmes flagged a lack of clear international recommendation for LTBI screening. One expert reported that their country had a high incidence of active TB (44/100,000 cases), so this was their focus. Three experts said they were positive about future LTBI screening of migrants but requested more data on effectiveness and emphasised the need to convince governmental bodies of the utility of LTBI screening.

### Current approaches to LTBI screening in migrants

**Figure 2:**
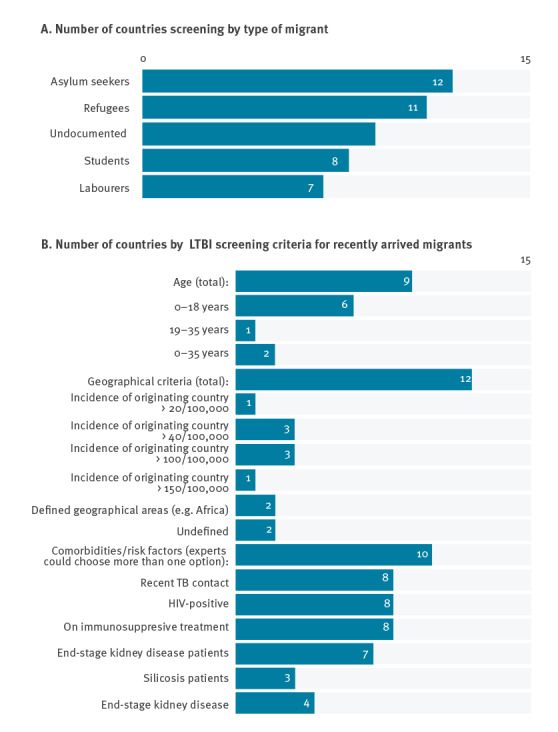
Category of migrants screened for latent tuberculosis infection (A) and criteria for latent tuberculosis infection screening programmes of recently arrived migrants (B), European Union/European Economic Area countries and Switzerland, September 2019–February 2020 (n = 15)

The majority of the experts in countries with LTBI programmes (12/15) reported screening of asylum seekers and/or refugees. Nine reported screening migrants who were undocumented, eight reported screening migrants who were students, and seven reported screening migrants who were labourers. Six experts reported screening all the above categories of migrants (Figure 2A).

Experts could choose more than one category.

Nine of 15 experts reported their countries performed LTBI screening of recently arrived migrants after settlement. Seven of 15 reported screening at the time of arrival (two experts reported performing screening at the time of arrival and after settlement) and one expert reported that their country screened migrants also before arrival using pre-departure screening programmes. Eight experts reported screening recently arrived migrants in either primary care or tertiary facilities (e.g., specialised TB centres), six reported screening migrants who were in refugee camps, and five reported screening migrants who were in specialist migrant centres or HIV centres.

Nine of the 15 experts reported screening on the basis of age: six reported screening solely on migrants under 18 years old, one reported screening migrants between 19 and 35 years of age, and two reported screening all migrants under 36 years of age. Ten experts reported screening migrants who had contact with active TB cases and eight reported screening migrants based on comorbidities, with all eight screening HIV positive individuals  and persons receiving immunosuppressive treatment (Figure 2B).

Twelve of the 15 experts reported screening based on country of origin, 10 reported screening based on TB incidence within the country of origin, and two reported screening based on defined geographical areas such as Africa, Asia and the Middle East. The threshold for defining high incidence countries of origin for presenting migrants varied between countries. We found that participating countries used a range of definitions for a high-burden country, ranging from 40 cases per 100,000 to 200 cases per 100,000 (Figure 2B).

### Diagnosis, treatment and follow-up approaches

Thirteen of 15 experts who reported existing LTBI screening programmes in their countries for migrants reported the use of either tuberculin skin tests (TST) and/or interferon gamma release assays (IGRAs) to screen for LTBI. Ten reported the use of x-rays in the diagnostic work-up. Diagnostic procedures differed by country and the approach depended on several factors, including the presence of Bacille Calmette-Guérin (BCG) scar (two experts), age (five experts), and area of work such as migrants with visas or migrants working in healthcare facilities (two experts).

In terms of treatment approach, 10 of 15 experts reported that their countries offered treatment to all migrants diagnosed with LTBI, with two only offering treatment to younger adults (under 40 years old and under 35 years old). All experts reported that their countries followed at least one of the following recommended regimens: rifampicin and isoniazid (3 months), rifampicin (4 months), or isoniazid (6 or 9 months). Seven experts reported the shortest treatment being the first option. One expert noted that rifampicin is expensive in their local context and therefore the isoniazid-based longer regimen is preferred.

Duration and frequency of follow-up varied across countries. Fourteen of the 15 experts reported that systems were in place to follow-up migrant patients, and all 15 performed liver enzyme tests to evaluate the potential liver toxicity of the drugs. However, they reported variations in time frames for follow-up, with six reporting monthly follow-up visits, one performing follow-up at week two and then at 1 month, and one performing follow-up at week two, four, and eight. A 24-month follow-up was performed in two countries, and seven offered counselling and/or peer-based support during treatment and follow-up.

Of the 15 experts who reported LTBI screening in recently arrived migrants, 12 reported that screening and treatment were free of charge. Of the three who reported on charges for LTBI screening and treatment, one reported that free LTBI screening and treatment was offered to migrants who lacked social security, one reported free LTBI screening and treatment only if the migrants were under 18 years old, and one reported that the treatment was free in their country, but screening was not.

### Migrant drop-out in LTBI screening programmes and current challenges

**Figure 3:**
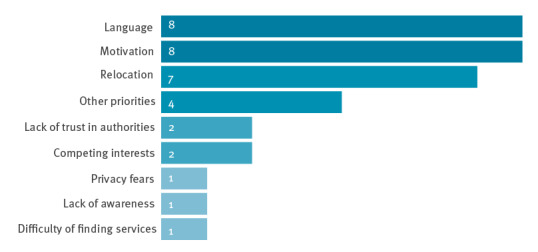
Reasons migrants dropped out of latent tuberculosis infection screening programmes, European Union/European Economic Area countries and Switzerland, September 2019–February 2020 (n = 15)

Six of 15 experts reported that migrants experience higher rates of drop-out in their view compared with the non-migrant population. Five reported no differences in drop-out rates and four reported that the situation was unclear or they were unaware of any differences. Eight experts reported that adherence to treatment for LTBI was the biggest challenge, and three experts noted that engaging migrants in screening was difficult, with a range of other factors identified (Figure 3). Reasons mentioned for drop-out were migrants’ lack of motivation and language barriers (n = 8), relocation (n = 7), and competing priorities of the migrants (n = 5), followed by a lack of trust in authorities (n = 2) (Figure 3).

Experts could choose more than one category.

### Views of experts on existing guidelines and current practice

Of the 15 experts, 14 reported using national guidelines, eight reported using WHO guidelines, and six reported using ECDC guidelines to determine who to screen. Eight experts reported several effective ways of engaging migrants in LTBI screening programmes. Responses ranged from building greater awareness among migrants about LTBI screening through leaflets and educational campaigns to providing materials in migrants’ languages. In addition, delivering educational campaigns to medical staff, government officials and the general public about the importance of screening migrants for LTBI was deemed important, and using technology (e.g., digital health or telemedicine technologies) to increase efficiency and dissemination and better tailoring of screening programmes specifically for migrants were also noted as ways to increase the number of migrants screened.

**Figure 4:**
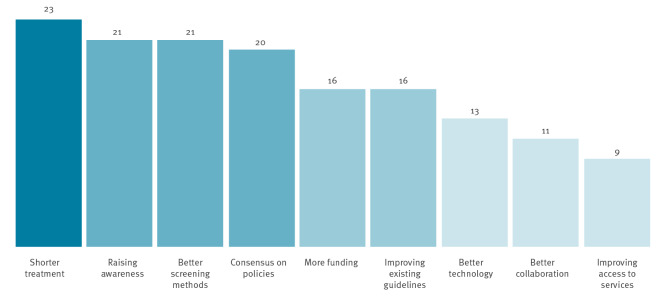
Priorities for strengthening design and delivery of latent tuberculosis infection screening programmes, European Union/European Economic Area countries and Switzerland, September 2019–February 2020 (n = 30)

Of the 30 experts who completed the survey, 22 reported that screening for LTBI, especially for migrants coming from high-incidence countries into low-incidence countries, should be expanded and performed at the point of entry or at the holding level. Ten stated that a focus should also be given to screening within the community, and two stated that screening should take place after relocation. The experts highlighted a range of priorities for strengthening the design and delivery of LTBI programmes for migrants in EU/EEA countries (Figure 4). The free-text responses highlight that the experts believe that it is important to expand current programmes to include all types of migrants, to invest in LTBI programmes for migrants and to use technological tools and equipment to streamline this process.

## Discussion

According to our survey, 15 of the 30 countries with responses in the EU/EEA and Switzerland operate an LTBI screening programme targeting migrants, and five countries plan to implement one in the near future. The majority of experts were keen to see a renewed focus on expanding efforts to screen for LTBI in migrants entering low-incidence countries. Screening programmes include mainly asylum seekers and refugees, with a focus on younger migrants (< 36 years old) and screen migrants after settlement in a range of settings. Most countries use country of origin as the main screening criteria, yet European countries use different criteria to define a high-burden TB country of origin, including from 40 cases per 100,000 to 200 cases per 100,000, originating from Asia, Africa or the Middle East, and age group. Countries took similar approaches to diagnosis but used one of three recommended treatment regimens and divergent approaches to follow-up. Six country experts from countries with LTBI screening programmes reported that migrants experience higher rates of drop-out compared with other groups for a range of reasons, suggesting that LTBI programmes may need to be strengthened to improve effectiveness and cost-effectiveness. Our data highlight a range of approaches to LTBI screening in migrants in EU/EEA countries and Switzerland and suggest that greater clarity and further evidence are needed about what constitutes an effective and cost-effective approach in order to reduce the number of active TB cases and meet regional and global elimination targets.

This work builds on previous research and discourse around approaches to screening migrants for LTBI. Kunst et al. [18], in a 2017 systematic review of past data and recent surveillance data from certain EU/EEA countries on active TB and LTBI, reported that countries use different definitions for coverage and yield of screening for active TB and LTBI and different screening strategies and settings. Therefore, they proposed the set up of a European platform for multi-country data collection and analysis, sharing of policies and practices among countries, and harmonisation of screening strategies for migrants. This work also builds on similar findings from a study of national-level European TB experts who focus on LTBI and active TB in refugees, where 19 of 38 experts reported systematic screening in-country for LTBI of migrants who were refugees. However, our study is broader as it focuses on a wider group of migrants [19].

In our study, for example, we report a range of thresholds being adopted across EU/EEA countries and Switzerland for screening based on country of origin, but in many cases the rationale supporting the screening approach remains unclear. Pareek et al. [7] reported that screening for latent infection can be cost effective, especially screening of individuals from countries with a TB incidence of more than 250 cases per 100,000 (incremental cost-effectiveness ratio (ICER) was 21,536 EUR per prevented case of TB) and a strategy of screening migrants from countries with more than 150 cases per 100,000 (including immigrants from the Indian subcontinent), which identified 92% of infected immigrants and prevented an additional 29 cases of active TB. Our study suggests that screening mainly or exclusively migrant groups such as asylum seekers and refugees may need to be reconsidered. In our survey, most countries screen asylum seekers and refugees; however, this group of migrants form only a small subset of the total migrant population within EU/EEA countries [20]. Furthermore, the top countries where migrants arrive in the EU/EEA are high-incidence TB countries, the proportion of TB among migrants has increased from 10% in 2000 to 25% in 2010, and the main reported reason for active TB among migrants in the EU is reactivation of LTBI, suggesting that screening a wider group of migrants is warranted [8,21].

The literature has shown that treatment adherence by migrants depends on many factors [4], a finding that is supported by our study. Experts reported that migrants have higher rates of drop-out than other groups because of a lack of motivation, competing interests, and language barriers. It is unclear whether divergent approaches to screening location and timing of screening after arrival play a part in drop-out. Guidelines [8,22,23] highlight the need for educational programmes, patient-centred LTBI management and free screening and treatment for migrants. Encouragingly, most countries in our survey reported free screening and treatment for LTBI.

Experts commented that they would like to see an expansion and strengthening of LTBI programmes targeting migrants. This could include encompassing a wider range of migrants and a broader age spectrum, offering migrants tailored advice (including translating materials that address migrant-specific needs) and support, and combining LTBI screening with other diseases and service delivery models – e.g., vaccination and other infectious disease screening programmes that address the specific needs of migrants. Migrants face many barriers to accessing healthcare and a range of socioeconomic, linguistic and cultural factors need to be considered. To improve such programmes will be critical for identifying community-based approaches that engage migrants in the design and delivery of interventions. Data are needed to show the effectiveness and cost-effectiveness of these programmes to support governments in decision making about the utility of such screening approaches.

A limitation of our study is that we asked national experts to reflect on current policies and practice, which implies that we might have missed some local efforts not yet implemented on a national level. In addition, we are aware that experts may have differing views and knowledge levels. Considerable efforts were made to identify the correct respondent for each country, to mitigate against this and to identify the expert either responsible or with direct knowledge of the current national TB/LTBI programme. If the expert initially approached was not directly involved in the national TB/LTBI programme, we identified the correct expert either through consultation with other experts within the country or by performing digital searches and contacting the appropriate expert.

### Conclusions

This survey provides policymakers and planners with information on the current state of LTBI screening approaches in EU/EEA countries and Switzerland. Our findings could inform national and local decision makers in how to strengthen and develop these programmes and improve health outcomes in migrant populations across the EU/EEA and Switzerland as we move towards TB elimination targets. Providing migrants with preventive health services, including migrants residing in high-income countries in the EU/EEA, is a core component of the universal health coverage agenda within the context of the 2030 Agenda for Sustainable Development and its associated goals.

## *Note

At the time of conducting this research, the United Kingdom was part of the European Union.

## Supplementary Data

302000 Supplementary Material 1

## Supplementary Data

67000 Supplementary Material 2
