# Supplementary material for: Country-specific approaches to latent tuberculosis screening targeting migrants in EU/EEA* countries: A survey of national experts, September 2019 to February 2020
Source: Euro Surveill. 2022 Mar 24;27(12):2002070. doi: 10.2807/1560-7917.ES.2022.27.12.2002070 (PMC8950856; doi:10.2807/1560-7917.ES.2022.27.12.2002070)
Supplement: Supplementary Material 1 [file 20-02070_MARGINEANU_Supplement_1.pdf]

Supplement 1 - Survey

This supplementary material is hosted by *Eurosurveillance* as supporting information alongside the article 'Country-specific approaches to latent tuberculosis screening targeting migrants in EU/EEA* countries: A survey of national experts, September 2019 to February 2020', on behalf of the authors, who remain responsible for the accuracy and appropriateness of the content. The same standards for ethics, copyright, attributions and permissions as for the article apply. Supplements are not edited by *Eurosurveillance* and the journal is not responsible for the maintenance of any links or email addresses provided therein.

ESCMID MANAGING INFECTIONS PROMOTING SCIENCE

ESGITM and ESGMYC survey of policy and practice for latent tuberculosis infection (LTBI) screening in migrant populations across Europe

We would value your input into a short survey to explore how EU/EEA countries approach LTBI screening in recently arrived migrants, on behalf of the ESCMID Study Group for Infections in Travellers and Migrants (ESGITM) and the ESCMID Study Group for Mycobacterial Infections (ESGMYC).

For the purposes of this survey, definitions are:

- Recently arrived migrant: Foreign-born, in the host country for <10 years
- Refugee: Granted asylum in the host country
- Asylum seeker: Awaiting a decision on their asylum application in host country

After completing the questionnaire, please return this form to: ismargineanu@gmail.com

Name: Click here to enter text.Email: Click here to enter text. Field of expertise: Click here to enter text.Position: Click here to enter text.

Country of employment: Choose an item.

Section 1: Current screening approach in recently arrived migrants

1. Do you perform latent tuberculosis infection (LTBI) screening in your country for any population group?
   ☐Yes.

   ☐No

2. Do you target recently arrived migrants for **LTBI** screening in your country?
   ☐Yes. **If Yes, please proceed to section 2.**

☐No, but there are plans to perform LTBI screening for recently arrived migrants in the near future. Please shortly describe these plans whilst keeping in mind Sections 2-4: Click here to enter text.

☐No. Could you briefly explain the reasons for the current situation: Click here to enter text.

If No, please proceed to section 5.

Section 2: Current LTBI screening approach in recently arrived migrants

3. Is LTBI screening mandatory for all recently arrived migrants?
   ☐Yes

   ☐No

4. Which categories of migrants do you screen for LTBI? [Select ***all*** that apply]

   ☐ All recently arrived foreign born nationals

   ☐Students ☐ Highly skilled labour migrants

   ☐Low-skilled labour migrants ☐Temporary workers

   ☐Refugees ☐Asylum seekers

   ☐Undocumented migrants

   ☐ Migrants who arrive through specific visa schemes, please define: Click here to enter text.

   ☐Other, please specify: Click here to enter text.

   ☐LTBI screening performed based on other criteria rather than category of migrant

5. If you screen on the basis of migrant country of origin what are your specific criteria? [Select ***all*** that apply]

**Burden of TB criterion:**	**Geographical region criterion:**
☐Migrants from **high** burden countries. Please define a high burden country: Click here to enter text. per 100.000 population<br>☐Migrants from **medium** burden countries. Please define a high burden country: Click here to enter text. per 100.000 population<br>☐Migrants from **low** burden countries. Please define a high burden country: Click here to enter text. per 100.000 population<br>☐Other definition regarding burden: Click here to enter text.	☐Migrants originating from the following geographical regions or countries (name all which apply): Click here to enter text.
☐Burden of TB in country of origin in **not** a criterion.	☐Geographical region of TB **not** a criterion.

6. If you screen on the basis of age, what are the specific age groups you target? [Select ***all*** that apply]
☐0-18 years ☐ 19-35 years ☐36-65 years ☐≥66 years ☐Age not a criterion.

7. If you screen based on comorbidities/other criteria, which specific comorbidities in recently arrived migrant groups do you target? [Select ***all*** that apply]

☐HIV infection
☐any Immunosuppressive treatment
☐ only TNF-alpha inhibitors
☐Pulmonary fibrosis
☐End stage kidney disease
☐ Diabetes Mellitus
☐ Silicosis
☐Recent known contacts of infectious TB cases
☐Transplant
☐Other, please specify: Click here to enter text.

8. When is LTBI screening performed in recently arrived migrants? [Select ***all*** that apply]
☐Before arrival

☐On arrival

☐After some time during settlement phase

☐Other, please specify: Click here to enter text.

If you have selected more than one option, please explain what percentage and which types of migrants (if applicable) are screened at different time points: Click here to enter text.

9. Where is LTBI screening performed? [Select ***all*** that apply]

☐ In refugee camps
☐In holding centres (reception centres, detention centres)
☐Within HIV services
☐ Within antenatal services
☐In primary care
☐In specialist (tertiary) care
☐In specialist migrant health centres
☐As part of a pre-entry assessment
☐Through the visa programme
☐At the workplace or school
☐In other medical facilities
☐Other, please specify: Click here to enter text.

Section 3: Diagnosis and management approaches to LTBI

10. What are the screening methods you use in your LTBI screening for recently arrived migrants? [Select ***all*** that apply]
    ☐History suggestive for TB exposure
    ☐TST ☐IGRA QuantiFERON ☐IGRA T-SPOT
    ☐X-Ray (modifications suggesting previous/healed TB)
    ☐Other, please specify: Click here to enter text.

11. Do these screening methods differ based on certain criteria? [Select ***all*** that apply]
    ☐Yes, based on age. In what way? Click here to enter text.
    ☐Yes, based on country of origin. In what way? Click here to enter text.
    ☐Yes, based on type/context of migrant. In what way? Click here to enter text.
    ☐Yes, based on other criteria. Please specify: Click here to enter text.
    ☐ yes, based on the history/evidence of scar of BCG-vaccination

12. If you detect LTBI in a migrant, what is the approach taken?
    ☐**All** migrants with positive LTBI test are offered preventive therapy
    ☐**Some** migrants with positive LTBI testing undergo preventive therapy. Please state who specifically: Click here to enter text.
    ☐ **Some** migrants with positive LTBI testing are followed and observed. Please state who specifically: Click here to enter text. Please state how long they are observed: Click here to enter text.
    ☐No migrants with positive LTBI testing are offered preventive therapy

13. Which preventive therapy regime would you chose for eligible persons?
    First choice: Drug Choose an item. for Click here to enter text. months
    Second choice: Drug Choose an item. for Click here to enter text. months
    Third choice: Drug Choose an item. for Click here to enter text. months
    ☐Other, please specify: Click here to enter text.

    If you have mentioned more than one choice for preventive therapy, how do you choose between the different preventive therapy treatment approaches? Click here to enter text.

14. Do you monitor a recently arrived migrant who began preventive therapy?
    ☐Yes, all recently arrived migrants are followed-up, for Click here to enter text. months
    ☐Yes, only in specific cases: Click here to enter text., for Click here to enter text. months
    ☐No

15. Do you keep a record of all recently arrived migrants who have been screened, treated, or followed-up for LTBI?

☐Yes, for **all** recently arrived migrants

☐Yes, for **some** recently arrived migrants

☐No, we do not keep records

16. In those eligible to preventive therapy, before starting the therapy [Select ***all*** that apply]:

☐ Liver tests are performed (i.e. transaminases, bilirubine)

☐ Tests for HCV and HBV are performed (HCV-Ab, HBsAg/HBsAb)?

17. In those undergoing preventive therapy, during therapy:

☐ Drug toxicity is monitored by liver tests (i.e. transaminases, bilirubine). Please describe the monitoring scheme: Click here to enter text.

18. IS the screening procedure and preventive therapy free of charge?:

☐ Yes

☐ No

☐Some aspects are free of charge: Click here to enter text.

19. In your country, do migrants experience higher rates of drop out compared to the host populations undergoing LTBI screening and treatment?

☐Yes (proceed to question 16)

☐No (proceed to section 4, question 18)

20. If Yes at question 19: in your opinion, on a national level, what key problems do migrant face with LTBI screening, treatment, or follow-up? [Select ***all*** that apply]

☐Engaging them in LTBI screening

☐Returning for their screening results

☐Initiating preventive therapy when they test positive for LTBI.

☐Adhering to preventive therapy

In your national screening programmes:

☐What percentage of migrants refuse treatment per annum Click here to enter text.

☐What percentage of migrants are lost to follow-up per annum Click here to enter text.

21. If yes at question 19, in your opinion what are the key issues that migrants face that make it more difficult for them to complete LTBI screening, treatment, or follow-up? [Select ***all*** that apply]

☐ relocation and housing issues

☐ trust issues with service providers

☐privacy fears

☐language barriers

☐other competing priorities

☐lack of motivation

☐high bureaucracy related to accessing screening

☐other reasons, please specify: Click here to enter text.

Section 4: Guidelines and practice

22. Are there specific national, regional, or international guidelines that you follow in your country for LTBI screening in recently arrived migrants?
☐Yes (proceed to question 23)

Please choose all that apply: ☐National guideline ☐ ECDC ☐ CDC ☐ IOM ☐WHO ☐Other, please specify: Click here to enter text.

☐No (proceed to question 25)

23. On a scale from 1 to 10 with 1 being “not at all important” and 10 being “very important”, how important are the guidelines you are using in your clinical decision-making process regarding LTBI screening in recently arrived migrants?
Choose an item: Choose an item.

24. Are you satisfied with the guidelines available in guiding your approach to LTBI screening in recently arrived migrants?
☐Yes

☐No: please explain why Click here to enter text.

Section 5: Next steps in delivery of LTBI screening to migrant populations

25. Are you aware of any innovative approaches in your country to **raise awareness of LTBI screening** (eg. educational campaigns, leaflets on arrival, outreach programmes) or **ensure capturing and adherence** of migrants in LTBI screening? Please describe them briefly here: Click here to enter text.

26. In your opinion, is it the right approach to expand LTBI screening programmes across the EU/EEA targeting recently arrived migrants?
☐Yes, short description of reasons: Click here to enter text.

☐No, short description of reasons: Click here to enter text.

27. In your opinion, at what stage in the migration process should LTBI screening be offered?

☐Pre-entry, in the countries of origin

☐At the point of entry

☐At the holding level (reception centres, refugee camps, detention centres)

☐Within the community after arrival eg, primary care

☐By employers/schools before or after arrival

☐Once residence status has been gained (eg. granted asylum)

☐Other, please specify: Click here to enter text.

28. In your view, what needs to change in terms of current approaches to LTBI screening programmes targeting recently arrived migrants in your country? Click here to enter text.

29. Please state if you believe any of these steps would be a priority to improving LTBI screening approaches targeting recently arrived migrants:

Step	Priority	Not a priority
Developing migrant specific LTBI guidelines	☐	☐
More regional/national funding for LTBI screening in migrants across EU/EEA region	☐	☐
Finding a better consensus on which specific recently arrived migrants should be targeted for LTBI screening on or after arrival	☐	☐
Better collaboration with the originating countries of dominant migrant groups to the EU/EEA	☐	☐
Improved awareness raising/education amongst migrant populations about the benefits of screening and treatment for LTBI	☐	☐
Investing in newer/better methods of LTBI screening	☐	☐
Improving access to existing/current LTBI screening methods		
Adopting/promoting shorter treatment options	☐	☐
Investing in newer technology or software to aid in the LTBI screening and management process	☐	☐
Other, please specify: Click here to enter text.		

On behalf of the ESGITM/ESGMYC we would like to thank you for completing this questionnaire.

St George's, University of London (Sally Hargreaves, Jon S Friedland)

University Medical Centrum Groningen (Ioana Margineanu, Ymkje Stienstra)

ESCMID MANAGING INFECTIONS PROMOTING SCIENCE

St George's University of London

rijksuniversiteit groningen

umcg

MARIE CURIE ALUMNI

Pronkjewail

Istituto Nazionale per le Malattie Infettive IRCCS L. Spallanzani
