# Supplementary material for: Country-specific approaches to latent tuberculosis screening targeting migrants in EU/EEA* countries: A survey of national experts, September 2019 to February 2020
Source: Euro Surveill. 2022 Mar 24;27(12):2002070. doi: 10.2807/1560-7917.ES.2022.27.12.2002070 (PMC8950856; doi:10.2807/1560-7917.ES.2022.27.12.2002070)
Supplement: Supplementary Material 2 [file 20-02070_MARGINEANU_Supplement_2.pdf]

Supplement 2: Institutions of participating experts

This supplementary material is hosted by Eurosurveillance as supporting information alongside the article ‘Country-specific approaches to latent tuberculosis screening targeting migrants in EU/EEA* countries: A survey of national experts, September 2019 to February 2020’, on behalf of the authors, who remain responsible for the accuracy and appropriateness of the content. The same standards for ethics, copyright, attributions and permissions as for the article apply. Supplements are not edited by Eurosurveillance and the journal is not responsible for the maintenance of any links or email addresses provided therein.

**Country**	**Institution**	**Area of Expertise**
Austria	Ministry of Health	Public Health/Infectious Diseases
Belgium	National Tuberculosis Association	Public Health/Respiratory Diseases
Bulgaria	National Tuberculosis Programme	Public Health/Tuberculosis in Children
Croatia	National Public Health Institute	Public Health/Epidemiology
Cyprus	National Public Health Institute	Infectious Diseases/Tuberculosis
Czech Republic	National Tuberculosis Programme	Epidemiology/Tuberculosis
Denmark	Infectious Diseases Surveillance Unit	Infectious Diseases/Tuberculosis
Estonia	National Tuberculosis Programme	Multi drug resistant tuberculosis
Finland	National Tuberculosis Programme	Tuberculosis Epidemiology/Surveillance/Control
France	National Tuberculosis Prevention Network	Tuberculosis
Greece	National Tuberculosis Hospital	Multi drug resistant tuberculosis
Iceland	National Tuberculosis Programme	Respiratory Diseases
Ireland	National Public Health Institute	Public Health
Italy	Ministry of Health, National Public Health Institute	Tuberculosis
Latvia	National Public Health Institute	Public Health
Lithuania	Ministry of Health	Respiratory Diseases

Luxembourg	National Infectious Diseases Control Unit	Infectious Diseases
Malta	National Infectious Diseases Control Unit	Infectious Diseases, Migrant Health
Netherlands	National Tuberculosis Programme	Public Health, Infectious Diseases
Norway	National Tuberculosis Programme	Respiratory Diseases
Poland	National Tuberculosis Programme	Microbiology
Portugal	National Tuberculosis Programme	Respiratory Diseases
Romania	National Tuberculosis Programme	Respiratory Diseases
Slovakia	National Tuberculosis Programme	Respiratory Diseases
Slovenia	National Tuberculosis Programme	Internal Medicine/Tuberculosis
Spain	National Tuberculosis Programme	Tuberculosis
Sweden	National Public Health Institute	Epidemiology, tuberculosis
Switzerland	National Tuberculosis Programme	Tuberculosis, Asylum seekers
Lichtenstein	National Tuberculosis Programme	Tuberculosis, Asylum seekers
United Kingdom	National Tuberculosis Programme	Tuberculosis
